# Sir Roy Calne, the Founding President of ESOT

**DOI:** 10.3389/ti.2024.12790

**Published:** 2024-02-23

**Authors:** Gabriel C. Oniscu

**Affiliations:** Division of Transplantation, Department of Clinical Science, Intervention and Technology, Karolinska Institutet (KI), Stockholm, Sweden

**Keywords:** in memoriam, in memoriam Roy Yorke Calne, ESOT, organ transplantation, Roy Yorke Calne

On 6th of January, the world of transplantation lost one of its last original pioneers. Sir Roy Calne was a giant of transplantation and his achievements are so numerous and so far reaching that no eulogy could capture his titanic contribution to the field.

The hallmark of Roy Calne’s career was innovation. He was a true clinician-scientist, who has shaped many aspects of the emerging field of transplantation, pushed the boundaries with novel surgical techniques, innovative types of transplants but also through cutting edge science. His particular interest in improving the outcome of transplantation and perseverance in pursuing better immunosuppressive therapies became the cornerstone of a new era in transplantation. As a result, transplantation emerged from the experimental stages into main stream clinical care and the field changed forever.

A true visionary, he recognised that making transplantation successful was not just about surgical advances but required collaborations across many disciplines and education of future generations of healthcare practitioners. He fostered this successfully in Cambridge, encouraged countless visitors to adopt this concept and shared his vision nationally and internationally.

It is this foresight and perseverance that in 1982 brought together all European healthcare practitioners under one umbrella that was to become the European Society for Organ Transplantation. It is worth quoting from the archives of ESOT as recorded by Dr Uhlschmid from Zurich: “Following the enthusiastic response from those participating in the Gelin-Memorial Symposium in Gothenburg in November 1981, it was felt that there was a need for a new society to be formed which would represent more accurately the aims and needs of transplantation surgery and surgeons in Europe. A number of European transplant surgeons met and formed a steering committee for the foundation of a society, the proposed name of which would be The European Society of Transplant Surgeons.” At the founding assembly which took place on 28th of April 1982, the identity of the new society was discussed, and it was Roy Calne who argued that this should not be yet another surgical society but should involve “all persons actively involved in organ transplantation.” The assembly approved the motion and ESTS became ESOT with Roy Calne elected as the first President of the new society.

As a Society we have come a long way since that inaugural assembly, but Roy Calne’s philosophy of multi-disciplinarity has remained the key pilar of ESOT to this day. Although he retired from clinical practice many years ago, he never stayed away from the society he helped to shape and was an active contributor to many Congresses, always happy to speak to new generations of transplanters and share his experience. In 2005, ESOT decided to award Honorary Memberships in recognition to contributions to the field and there was no more appropriate recipient for this accolade other than Roy Calne together with his friends and fellow pioneers Tom Starzl and Rene Küss.

Over the last 40 years, each ESOT President has had the challenging but rewarding task of building on the legacy of our forebearers and keeping our Society at the forefront of innovation in science and clinical care. This is not an easy mission considering the tall order set by Roy Calne, the Founding President of ESOT!

Sir Roy’s passing marks the end of an era but his legacy lives on through countless generations whom he inspired and a robust and visionary Society that he has nurtured.

## Data Availability

The original contributions presented in the study are included in the article/Supplementary material, further inquiries can be directed to the corresponding author.

